# The New Repertory

**Published:** 1885-05

**Authors:** 


### THE NEW REPERTORY.

In the April issue of this journal a circular was issued setting forth the character and purpose of the Repertory of Characteristics which is now being revised for the press. The editor desires to return his sincere thanks for the many offers of assistance he has received; also for the kind words of encouragement and numerous subscriptions sent him. In *two* weeks about one-third of the desired number of subscriptions were received. We still need subscriptions, and would call attention to the new circular which accompanies this number. Ask your friends to subscribe.

